# The story of critical care in Asia: a narrative review

**DOI:** 10.1186/s40560-021-00574-4

**Published:** 2021-10-07

**Authors:** Jason Phua, Chae-Man Lim, Mohammad Omar Faruq, Khalid Mahmood Khan Nafees, Bin Du, Charles D. Gomersall, Lowell Ling, Jigeeshu Vasishtha Divatia, Seyed Mohammad Reza Hashemian, Moritoki Egi, Aidos Konkayev, Mohd Basri Mat-Nor, Gentle Sunder Shrestha, Madiha Hashmi, Jose Emmanuel M. Palo, Yaseen M. Arabi, Hon Liang Tan, Rohan Dissanayake, Ming-Cheng Chan, Chairat Permpikul, Boonsong Patjanasoontorn, Do Ngoc Son, Masaji Nishimura, Younsuck Koh, for the Asian Critical Care Clinical Trials Group

**Affiliations:** 1 FAST and Chronic Programmes, Alexandra Hospital, National University Health System, Singapore, Singapore; 2 Division of Respiratory and Critical Care Medicine, Department of Medicine, National University Hospital, National University Health System, Singapore, Singapore; 3 Department of Pulmonary and Critical Care Medicine, Asan Medical Center, University of Ulsan College of Medicine, Seoul, South Korea; 4 General Intensive Care Unit, Emergency and COVID ICU, United Hospital Ltd, Dhaka, Bangladesh; 5 Ministry of Health, Department of Critical Care Medicine, RIPAS Hospital, Bandar Seri Begawan, Brunei Darussalam; 6 State Key Laboratory of Complex Severe and Rare Diseases, Medical ICU, Peking Union Medical College Hospital, Beijing, China; 7 Department of Anaesthesia and Intensive Care, The Chinese University of Hong Kong, Prince of Wales Hospital, Hong Kong, China; 8 Department of Anaesthesia, Critical Care and Pain, Tata Memorial Hospital, Homi Bhabha National Institute, Mumbai, India; 9 Chronic Respiratory Diseases Research Center, National Research Institute of Tuberculosis and Lung Diseases, Shahid Beheshti University of Medical Sciences, Tehran, Iran; 10 Department of Anesthesiology and Intensive Care Medicine, Kobe University Hospital, Kobe, Japan; 11 Anaesthesiology and Reanimatology Department, Astana Medical University, Astana, Kazakhstan; 12 Anaesthesia and ICU Department, Institution of Traumatology and Orthopedics, Astana, Kazakhstan; 13 Department of Anaesthesiology and Intensive Care, International Islamic University Malaysia, Kuantan, Malaysia; 14 Department of Anaesthesiology, Tribhuvan University Teaching Hospital, Maharajgunj, Kathmandu, Nepal; 15 Department of Critical Care Medicine, Ziauddin University, Karachi, Pakistan; 16 Acute and Critical Care Institute, The Medical City, Pasig City, Philippines; 17 King Saud Bin Abdulaziz University for Health Sciences, King Abdullah International Medical Research Center, Ministry of National Guard Health Affairs, Riyadh, Kingdom of Saudi Arabia; 18 Mount Elizabeth Novena Hospital, Singapore, Singapore; 19 Department of Intensive Care Medicine, Gosford Hospital, Gosford, NSW Australia; 20 Section of Critical Care and Respiratory Therapy, Department of Internal Medicine, Taichung Veterans General Hospital, Taichung, Taiwan; 21 College of Science, Tunghai University, Taichung, Taiwan; 22 Department of Medicine, Siriraj Hospital, Mahidol University, Bangkok, Thailand; 23 Pulmonary and Critical Care Medicine, Department of Medicine, Faculty of Medicine, Khon Kaen University, Khon Kaen, Thailand; 24 Critical Care Unit, Center for Emergency Medicine, Bach Mai Hospital, Hanoi, Vietnam; 25 Aizenbashi Hospital, Osaka, Japan

**Keywords:** Critical care, Asia, Intensive care unit, Epidemiology, Culture

## Abstract

**Background:**

Asia has more critically ill people than any other part of our planet. The aim of this article is to review the development of critical care as a specialty, critical care societies and education and research, the epidemiology of critical illness as well as epidemics and pandemics, accessibility and cost and quality of critical care, culture and end-of-life care, and future directions for critical care in Asia.

**Main body:**

Although the first Asian intensive care units (ICUs) surfaced in the 1960s and the 1970s and specialisation started in the 1990s, multiple challenges still exist, including the lack of intensivists, critical care nurses, and respiratory therapists in many countries. This is aggravated by the brain drain of skilled ICU staff to high-income countries. Critical care societies have been integral to the development of the discipline and have increasingly contributed to critical care education, although critical care research is only just starting to take off through collaboration across groups. Sepsis, increasingly aggravated by multidrug resistance, contributes to a significant burden of critical illness, while epidemics and pandemics continue to haunt the continent intermittently. In particular, the coronavirus disease 2019 (COVID-19) has highlighted the central role of critical care in pandemic response. Accessibility to critical care is affected by lack of ICU beds and high costs, and quality of critical care is affected by limited capability for investigations and treatment in low- and middle-income countries. Meanwhile, there are clear cultural differences across countries, with considerable variations in end-of-life care. Demand for critical care will rise across the continent due to ageing populations and rising comorbidity burdens. Even as countries respond by increasing critical care capacity, the critical care community must continue to focus on training for ICU healthcare workers, processes anchored on evidence-based medicine, technology guided by feasibility and impact, research applicable to Asian and local settings, and rallying of governments for support for the specialty.

**Conclusions:**

Critical care in Asia has progressed through the years, but multiple challenges remain. These challenges should be addressed through a collaborative approach across disciplines, ICUs, hospitals, societies, governments, and countries.

**Supplementary Information:**

The online version contains supplementary material available at 10.1186/s40560-021-00574-4.

## Background

There is no one concept of Asia. This term is a diverse mix of histories, geographies, cultures, economies, and populations. Today, Asia is often seen as the land mass separated from Europe by an arbitrary line joining the Urals with the Caucasus and the Black Sea [1]. It comprises East Asia (the Far East), Southeast Asia (the East Indies and Indochina), West Asia (the Middle East), North Asia (Siberia), South Asia (the Indian subcontinent), and Central Asia (the ‘stans), and a whole host of low-, middle-, and high-income countries as defined by the World Bank according to gross national income per capita (Additional file 1) [2, 3]. It is home to 60% of the world’s population, and more critically ill people than any other part of our planet [4].

Critical care started—haltingly—across Asia in the 1960s. In this narrative review, through a lens that recognises the heterogeneity that defines the continent, we will discuss the development of the specialty, critical care societies and education and research, the epidemiology of critical illness as well as epidemics and pandemics, accessibility and cost and quality of critical care, culture and end-of-life care, and future directions for critical care in Asia. To write this review the co-authors, all prominent intensivists from the Asian Critical Care Clinical Trials (ACCCT) Group who represented 19 countries and regions, some of whom are current or past Presidents of their national critical care societies, first provided qualitative input through a questionnaire (Additional file 2). Selected sections of the original text were featured in a recently published article commemorating the Society of Critical Care Medicine’s 50th anniversary “International critical care—from an indulgence of the best funded health care systems to a core need for the provision of equitable care” [5]. The manuscript has since been substantially expanded and updated for both breadth and depth (Additional file 3 provides information on the multiple topics added).

### Critical care as a specialty

**Fig. 1:**
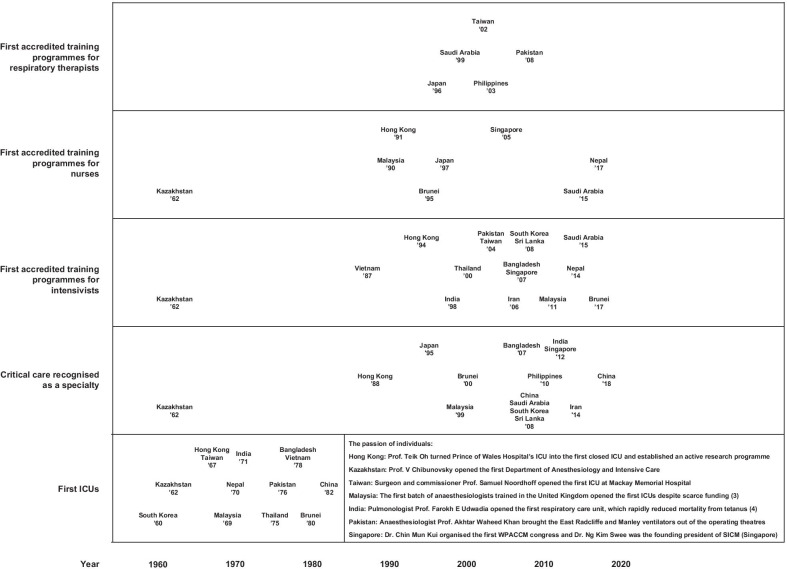
Timeline of the development of critical care. *ICU* intensive care unit

The first Asian ICUs surfaced in the 1960s and 1970s, after the poliomyelitis epidemic in Copenhagen started the era of mechanical ventilation in 1952 [6], and largely due to the passion of individuals [7, 8] (Fig. 1). However, it was not until the 1990s that specialisation in critical care began to take root. Obstacles to the formation of the specialty included the divide between pulmonologists and anaesthesiologists, and some countries like Nepal and Pakistan still do not recognise critical care as a distinct specialty. National accredited training programmes for intensivists took decades to materialise, with some starting small through local universities, such as in Nepal and Thailand. According to the Asian ICUs Structure and Process (AISP) study of 335 ICUs in 20 Asian countries, critical care fellowship programmes existed in 34%, 65%, and 67% of ICUs in low-, middle-, and high-income countries, respectively, in 2013–2014 (Additional file 4) [9].

Close to two-thirds of ICUs in the AISP study used a closed ICU operation model, in which only intensivists have admitting privileges [9]. However, there remains a shortage of intensivists in many countries. In Bangladesh, which needs at least 600 intensivists, only 30 doctors have received a postgraduate degree in critical care medicine. This lack is partly due to an absence of financial incentives to take on the specialty. In Pakistan, an anaesthesiologist earns ten times more in the operating theatre and a pulmonologist generates much more revenue from clinics and procedures than in ICUs.

Many countries still do not have accreditation programmes for critical care nursing and respiratory therapists (Fig. 1). Critical care nurses are generally in short supply, with approximately 18% of ICUs in low- and middle-income countries and 14% of ICUs in high-income countries in the AISP study having a nurse-to-bed ratio of 1:3 or leaner (Additional file 4) [9]. This is further aggravated by the brain drain of skilled ICU staff from low- and middle-income countries in the continent to high-income countries in Europe and North America. Meanwhile, 28%, 32%, and 53% of ICUs had respiratory therapists in low-, middle-, and high-income countries, respectively [9], with the Philippines being a large source of respiratory therapists worldwide [10]. Half of the ICUs had physiotherapists, dieticians, and clinical pharmacists, and one-third had social workers. However, only one-third and one-fifth of ICUs in low- and middle-income countries had clinical pharmacists and social workers, respectively [9].

Life as a healthcare worker in Asian ICUs is hard. Approximately half of physicians and nurses working in 159 ICUs across 16 Asian countries and regions suffered from burnout in 2015–2016 [11]. The prevalence of burnout varies across countries, with China reporting a rate of 82% among its intensivists in 2017–2019 [12].

### Critical care societies

**Fig. 2:**
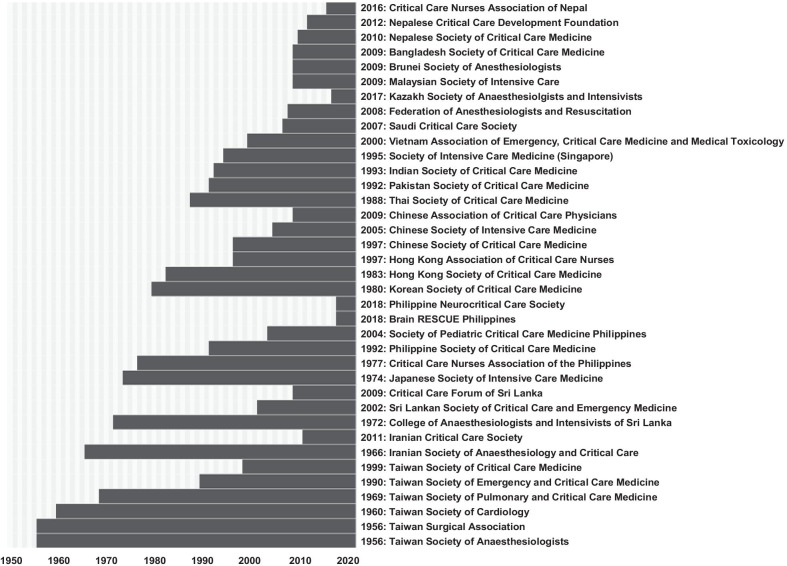
Founding year of selected critical care and affiliated societies Noteworthy details: The Japanese Society of Intensive Care Medicine is one of the oldest societies dedicated solely to critical care. The Korean Society of Critical Care Medicine played key roles in national critical care crises, including a surge of humidifier-disinfectants-induced lung injuries in 2011 [13]. The Indian Society of Critical Care Medicine was founded by a few enthusiastic consultants and has close to 12,000 members now

Critical care societies are integral to the field’s evolution in Asia (Fig. 2) [13]. Several of these were started as anaesthesiology societies in the 1950s and 1960s. Many are council members of the Asia Pacific Association of Critical Care Medicine (APACCM), which got its name in 2004, but originally came to being as the Western Pacific Association of Critical Care Medicine (WPACCM) in Tokyo in 1980. Many are also members of the World Federation of Intensive and Critical Care (WFICC) (Additional file 5).

### Critical care education

Beyond accreditation programmes and on-the-job training for ICU healthcare workers at the local and national level, multiple critical care scientific congresses exist in Asia, often in partnership between Asian societies and international ones (Table 1). The last time Asia hosted the World Federation’s World Congress of Intensive and Critical Care was in 2015, in Seoul. India will host the 17th World Congress in 2025. The Society of Critical Care Medicine (SCCM)’s Multi-professional Critical Care Review Course (MCCRC) is held in partnership with China, Japan, and South Korea, while its Fundamental Critical Care Support Course (FCCS) is held in countries like India, Iran, Saudi Arabia, and Singapore.

**Table 1:** Selected critical care congresses

National congresses	
Bangladesh	International Conference on Critical Care Medicine (CRITICON)
China	Chinese Association of Critical Care Physicians/Chinese Society of Critical Care Medicine: China Critical Care Congress
	Hong Kong Society of Critical Care Medicine Annual Scientific Meeting
India	Annual Conference of Indian Society of Critical Care Medicine (CRITICARE)
Iran	International Congress of Anesthesiology and Critical Care
	International Congress on Critical Care
Japan	Annual Meeting of the Japanese Society of Intensive Care Medicine
Malaysia	Annual Scientific Meeting on Intensive Care (ASMIC)
Nepal	National Conference of Nepalese Society of Critical Care Medicine
	International Conference of Critical Care Nurses Association of Nepal
Pakistan	International Conference on Anaesthesia, Pain and Intensive Care (APICON)
Philippines	Joint Annual Critical Care Convention of the Philippine Society of Critical Care Medicine and Society of Pediatric Critical Care Medicine Philippines
Saudi Arabia	Annual International Conference of the Saudi Critical Care Society
South Korea	Korean Society of Critical Care Conference Annual Congress
Sri Lanka	Sri Lankan Society of Critical Care and Emergency Medicine Annual Scientific Conference
Taiwan	Annual Joint Congress of Taiwan Society of Critical Care Medicine and Taiwan Society of Emergency and Critical Care Medicine
	Annual Congress of Taiwan Society of Pulmonary and Critical Care Medicine
Thailand	Critical Care Conference in Thailand
Vietnam	Annual Congress of Vietnam Association of Emergency, Critical Care Medicine and Medical Toxicology
Joint International Congresses	
Afghanistan Bangladesh Bhutan India Maldives Nepal Pakistan Sri Lanka	South Asian Association for Regional Cooperation Critical Care Societies Congress
Japan South Korea	Joint Congress of Korean Society of Critical Care Medicine—Japanese Society of Intensive Care Medicine
Japan Thailand	Joint Congress of Japanese Society of Intensive Care Medicine—Thai Society of Critical Care Medicine
Singapore Australia New Zealand	Asia Pacific Intensive Care Forum (SICM × ANZICS)
South Korea Taiwan	Joint Congress of Korean Society of Critical Care Medicine and Taiwan Society of Critical Care Medicine/Taiwan Society of Emergency and Critical Care Medicine
Taiwan ESICM	EuroAsia Conference
APACCM	APACCM Annual Scientific Meeting in partnership with national critical care societies

*ANZICS* Australian and New Zealand Intensive Care Society, *APACCM* Asia Pacific Association of Critical Care Medicine, *ESICM* European Society of Intensive Care Medicine, *SICM* Society of Intensive Care Medicine (Singapore)

Other educational platforms exist beyond traditional society structures. The Basic Assessment and Support in Intensive Care (BASIC) Collaboration has run courses in over 50 countries across the planet since 2004. The Asia Ventilation Forum (AVF) has conducted multiple lectures and workshops in numerous countries, including several from resource-limited settings, since 2006.

### Critical care research

An editorial in the inaugural issue of WPACCM’s journal, *Critical Care & Shock*, in 1998 wrote that “departments in our region have heavy clinical and teaching commitments that have left little time and opportunity for research” [14]. Two decades on, the community faces the same challenges. Pakistan, for example, has only one trained intensivist per 82 ICU beds in its teaching hospitals. There is a dearth of champions to systematically build a robust research culture. Between 1977 and 2013, only 72 articles from India, the world’s second largest country, were published in three major critical care journals [15].

There may be some light at the end of the tunnel. A sizeable minority of countries, such as China, Japan, Malaysia, Pakistan, Saudi Arabia, Singapore, South Korea, Taiwan, and Thailand, now have multicentre research groups. Some have national critical care journals. The Pakistan Registry of Intensive CarE (PRICE) today provides continuous information for service improvement and research [16]. The China Critical Care Clinical Trials Group was formed in 2009 to boost research in the world’s largest nation [17]. The Japanese and Koreans have co-created the Japan–Korea Intensive Care Study (JAKOICS) Group [18, 19]. The Saudi Critical Care Trials Group (SCCTG) has produced several high-impact papers [20–22]. The BASIC Clinical Research Course has been conducted in places like Hong Kong, Pakistan, Saudi Arabia, and Singapore. To build capacity in research coordinators, a new BASIC Clinical Research Coordination course was launched in collaboration with the SCCTG in Saudi Arabia in 2019.

In addition, the reach of research led by the European Society of Intensive Care Medicine (ESICM) Trials Group, the Australian and New Zealand Intensive Care Society (ANZICS) Clinical Trials Group, the Canadian Critical Care Trials Group (CCCTG), and WFICC has extended to Asia [23–30]. Significantly, after an inaugural multinational effort on the Management Of Severe sepsis in Asia’s Intensive Care unitS (MOSAICS) study [31], the ACCCT Group was formed in 2012 and has since conducted studies in 28 Asian countries and regions [9, 11, 32–36], some of which have also published their own national data from these studies [37–41].

### Epidemiology of critical illness

While the Global Burden of Disease study does not focus on ICUs per se, its 2015 edition provides an indication of the diseases that result in critical illness in Asia [42]. Ischaemic heart disease was the leading cause of premature mortality, measured as years of life lost (YLLs), in East and Southeast Asia and Oceania. Lower respiratory tract infections were among the ten leading causes of premature mortality in many countries; YLLs from these infections was higher than expected after adjustment for the socio-demographic index for Malaysia and Laos. Tuberculosis resulted in YLLs at least four times higher than expected in Indonesia and the Philippines.

On a related note, the Global Burden of Disease 2017 study found, despite a decreasing incidence over time, an estimated 22.8 million cases of sepsis in Asia in 2017, accounting for 47% of all cases worldwide [43]. While diarrhoeal disease was the commonest cause of sepsis, deaths were more often due to lower respiratory tract infections. High age-standardised mortality rates were especially seen in South Asia.

Narrowing down to critical care, 1928 patients from 127 ICUs in 12 Asian countries and regions were studied in the Intensive Care Over Nations (ICON) audit on the burden of critical illness in 2012 [30]. The mean age was 57 years, and the mean Acute Physiology and Chronic Health Evaluation (APACHE) II score 16.4. Mechanical ventilation was provided for 46%, and kidney replacement therapy for 12%. The median ICU length of stay was 4 days in East and Southeast Asia and 2 days in South Asia, and the overall hospital mortality was 19%. Adjusted hospital mortality was higher in countries with lower national income. Sepsis accounted for 27% of the cases.

In the 24-h point prevalence Extended Study on Prevalence of Infection in Intensive Care (EPIC) III study conducted in 2017, 60% of 3150 patients in 217 ICUs across 25 countries in Asia and the Middle East had suspected or proven infection [44]. Among them, 47% were community-acquired while the rest were nosocomial, 70% had a respiratory source, and 64% were culture-positive, of which three-quarters were Gram-negative bacteria. Multidrug resistance was seen for 17%, 39%, 55%, and 74% of all Pseudomonas, Escherichia coli, Klebsiella, and Acinetobacter infections, respectively. Similarly high rates of multidrug resistance were seen in local and national-level ICU studies, such as in Bangladesh, India, Nepal, and Vietnam [45–48]. Beyond the common bacteria that cause sepsis, pathogens less frequently seen in the West contribute to a substantial burden of disease in parts of Asia. A study of 1578 hospitalised patients with sepsis from 2013 to 2015 found that 8% had dengue, 6% had leptospirosis, and 6% had rickettsial infections [49].

Multinational and national-level ICU registries are rare in Asia, and include CRIT CARE Asia, the Japanese Intensive care PAtient Database (JIPAD), the Malaysian Registry of Intensive Care (MRIC), Sri Lanka’s National Intensive Care Surveillance (NICS) registry, the Pakistan Registry of Intensive Care (PRICE), and Singapore’s National Intensive Care Unit Repository (NICUR) [16, 50–53]. National-level studies outside of registries are uncommon [54–56]. The mix of ICU admissions depends on each country’s unique circumstances. For example, suicidal and accidental poisoning by household products, agricultural pesticides, and industrial chemicals, and trauma from road traffic accidents and gunshot wounds are common in India and Pakistan [57, 58].

### Epidemics and pandemics

It is worth remembering that Asia has seen its fair share of epidemics and pandemics throughout history, from the bubonic plaque to cholera to influenza [59, 60]. In the last century, the 1918–1919 Spanish H1N1, the 1957–1958 Asian H2N2, and the 1968–1970 Hong Kong H3N2 influenza pandemics together killed tens of millions of Asians [59, 61, 62]. It is, however, the coronaviruses that are more clearly etched in the collective memories of the Asian critical care community today. The severe acute respiratory syndrome (SARS) epidemic of 2002–2003 killed 729 people (including healthcare workers) in Asia, with China, Hong Kong, Taiwan, and Singapore, being the most affected [63]. More than half of the patients who required ICU admission died [64]. The Middle East respiratory syndrome (MERS) which was first described in 2012 has killed at least 780 people in Saudi Arabia [65], with a mortality rate of ICU patients of 68% [22]. South Korea was also hit hard by MERS [66].

### Coronavirus disease 2019 (COVID-19)

The lessons learnt from these earlier outbreaks helped some Asian ICUs deal with COVID-19—centres that had lived through the SARS and MERS outbreaks found that they were better prepared because of the ready availability of airborne infection isolation rooms, increased familiarity with hygiene measures and personal protective equipment, previous protocols for team segregation and staff rostering, existing processes for visitor screening and management, and a deep understanding of the importance of internal communication [67]. Indeed, the ACCCT Group rapidly gathered intensivists across the region to publish some of the earliest recommendations for the critical care management of COVID-19 [35]. Even then, the pandemic killed half of the critically ill in China and pushed the critical care capacity of its epicentre in Wuhan to its limits in early 2020 [68–70]. One year later, international media recurrently featured harrowing scenes of desperate families clinging onto severely stretched healthcare resources with overwhelmed ICUs, oxygen shortages, and a scarcity of trained staff during the pandemic’s second wave in India, a dark 3.5-month period that is estimated to have cost 1.4–2.4 million lives [71, 72]. Many other countries, especially in South Asia and Southeast Asia, were not spared [73]. By early August 2021, according to official figures which severely under-estimate reality, the SARS coronavirus 2 (SARS-CoV-2) had infected more than 50 million people across Asia [74]. Clearly, the risk of COVID-19 in Asia is not over, with the delta variant and other new variants of concern being a constant threat.

### Accessibility and cost of critical care

While some countries have sped ahead in the growth of ICUs, others, especially those in resource-limited settings, have taken decades to gain momentum. Nepal’s first ICU, a 5-bedded mixed unit, was established in 1970 at Bir Hospital, Kathmandu, and remained the country’s only ICU for almost 20 years. While there were cumulatively 3.6 critical care beds per 100,000 population across 23 countries and regions in 2015–2018, the median number of beds per 100,000 population varied from 2.3 to 4.6 to 12.3 in low- and lower-middle-, upper-middle-, and high-income countries, respectively (Additional file 6) [34]. Taiwan and Saudi Arabia have 28.5 and 22.8 critical care beds per 100,000 population, respectively, while Myanmar and Bangladesh have 1.1 and 0.7, respectively [34]. The 41-fold difference in capacity between the most-resourced and least-resourced countries suggests major differences in rationing of resources, with many critically ill patients being managed outside of ICUs in some countries even before COVID-19 struck.

Another barrier to accessibility to critical care is cost. In the Asian Collaboration for Medical Ethics (ACME) study of 1465 ICU physicians from 466 ICUs in 16 Asian countries and regions, those from low- and middle–income economies (especially China, the Philippines, and Bangladesh) were also more likely to accede to families’ requests to withdraw life-sustaining treatments in a patient with an otherwise reasonable chance of survival, so as to avoid further medical bills, than those from high-income economies [33]. Most healthcare systems in Asia provide individuals with little financial risk protection, and out-of-pocket payments for critical care can be catastrophic, leading to impoverishment for patients and families, especially those in low- and middle-income countries [75, 76].

### Quality of critical care

**Fig. 3:**
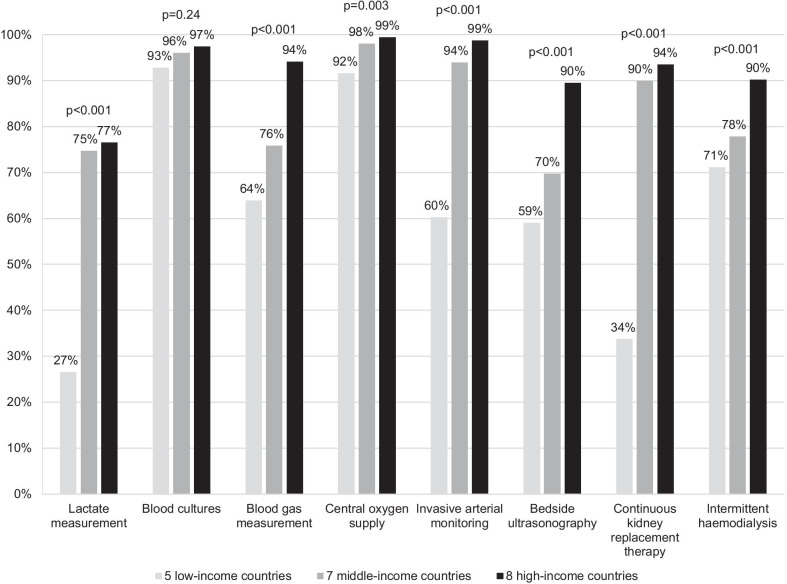
Proportion of ICUs capable of selected investigations and treatment. ICU, intensive care unit. Data from the Asian ICUs Structure and Process (AISP) study of 335 ICUs in 20 Asian countries, conducted between 2013 and 2014 [9]. Countries are categorised according to the World Bank income classification. *p*-values refer to unadjusted statistical comparisons using the Chi-square test. This survey involved many large government referral hospitals, and it is likely that smaller ICUs scattered across low-incomes countries are even more resource-limited

Multiple factors, including the training and availability of staff, the seamlessness and consistency of processes, and the readiness and reliability of infrastructure, affect quality of care. The AISP study found that many ICUs in low- and middle-income countries had limited capability for investigations and treatment (Fig. 3). Between two-thirds and three-quarters of ICUs had written protocols for admission and discharge criteria, sepsis, acute respiratory distress syndrome, sedation and analgesia, glucose control, venous thromboembolism prophylaxis, and weaning from mechanical ventilation [9]. Protocol or lack thereof, a survey done in 2016–2017 found substantial differences in weaning practices among 2,074 ICU physicians from 20 Asian countries and regions, with 53% frequently always using intermittent spontaneous breathing trials with pressure support in between, 44% frequently always using pressure support alone, and 20% frequently always using synchronised intermittent mandatory ventilation [36].

The PRactice of VENTilation in Middle-income Countries (PRoVENT-iMiC) and the MOSAICS studies give some useful insights into the quality of care provided, albeit through the relatively narrow lens of mechanical ventilation and sepsis, respectively [31, 77]. In the former, 33% of patients with acute respiratory distress syndrome in 54 ICUs across 10 countries were ventilated with a tidal volume of greater than 8 mL/kg predicted body weight in 2017–2018. In the latter, only 18% of patients with sepsis in 150 ICUs across 16 countries and regions—and 5% of patients in low-income countries—were treated with the Surviving Sepsis Campaign’s resuscitation bundle in 2008. Whether quality of care has improved since remains to be seen, but data from recent multicentre studies performed in selected countries give reason for optimism [78, 79]. Impressively, a national multifaceted quality improvement programme conducted in ICUs in 586 Chinese hospitals was associated with not just greater compliance to the sepsis bundle, but a decrease in ventilator-associated pneumonia rates between 2016 and 2018 [79].

### Culture and end-of-life-care in critical care

High-quality critical care must include high-quality end-of-life care. In the ACME study, while 70% of ICU physicians reported almost always or often withholding, 21% reported almost always or often withdrawing life-sustaining treatments for patients with no real chance of recovering a meaningful life [32]. There was however a wide range of answers across the 16 participating countries and regions, underscoring differences in the cultures of the many societies on this large continent [32, 37]. Interestingly, when the issue of financial burden to families was not considered, physicians in low- to middle-income countries were less likely to pronounce DNR than those in high-income ones, suggesting differences in values across the continent [32].

An issue that frequently surfaces in discussions on end-of-life care is patient autonomy, a concept strongly valued in the West [80]. On the other hand, intra-continent heterogeneity notwithstanding, the opinions of the family is key in many Asian cultures [81, 82]. Yet, more than half of the ICU physicians in the ACME study expressed discomfort discussing limitation of life-sustaining treatments with families, and more than one-third felt that families’ requests were almost always or often inappropriate [32].

### Future directions

**Fig. 4:**
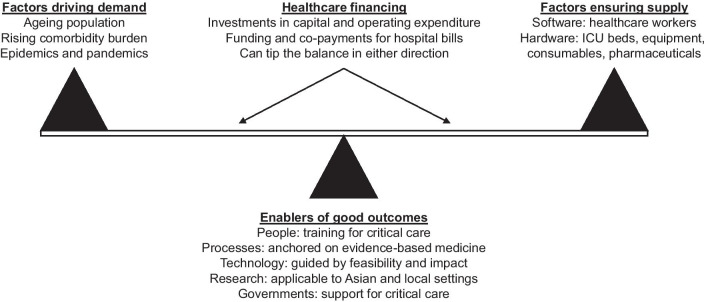
Balancing demand and supply while improving outcomes for critical care. *ICU* intensive care unit

Although critical care in Asia has seen its fair share of twists and turns this century and the last, some of these are not unique—they also apply to other continents, especially those with developing countries, such as Africa and South America. Just like much of the rest of the world, it is reasonable to say that Asian critical care is now at a crossroads, with a risk of worsening mismatch between demand and supply (Fig. 4). While some countries will see a rise in population as others see a fall (a decrease in population size by more than 25% by the year 2100 due to low total fertility rates is expected in 12 countries [83]), most of Asia will continue to age rapidly. The ageing population with its attendant comorbidity burden will impose severe demands for ICU beds, even as the shrinking workforce in many areas aggravates serious shortages of healthcare workers. More immediately, the COVID-19 pandemic has substantially increased the pressure on all countries to review their critical care capacity, enhance their pandemic preparedness, and rethink the very nature of their ICUs [35, 84–86]. The complexity of this exercise and the impossibility of a one-size-fits-all approach become painfully clear when one considers that while most if not all countries are facing ballooning healthcare costs, and that some continue to struggle with bare-minimum healthcare, let alone resource-intensive critical care [87]. Clearly, optimal matching of needs and resources will require healthcare financing policies that allow adequate investments in capital and operating expenditure, and appropriately balance funding and co-payments for hospital bills.

Several enablers of good outcomes must be considered in any discussion of critical care capacity. First, a passionate pursuit of training for critical care staff, especially in low- and middle-income countries, is crucial. Critical care societies and education networks can and must help through the creation of curriculum and the organisation of standardised short courses which may be conducted both in-person and virtually. Second, a relentless focus on evidence-based clinical processes, with alignment among healthcare workers within an ICU, is required. Agreement on quality indicators at a local, national, or international level will help improve adherence to these processes. Third, a nuanced investment in technology, guided by current feasibility and potential impact, is needed. While resource-limited countries must focus on getting the basics such as the availability of oxygen and ventilators right, technologically savvy ones such as China, Japan, Singapore, South Korea, and Taiwan, have the opportunity to harness the strengths of telemedicine, robotics, and artificial intelligence in the ICU. Fourth, a decisive push for high-quality research applicable to Asian and local settings, facilitated by integrated research infrastructure, networks, and registries, is important. The generation of new knowledge must then facilitate the practice of personalised critical care medicine that is tailored to Asia’s diverse populations and sensitive to its myriad cultures [32]. Finally, a clear mandate by governments to further develop critical care, complete with financial, policy, and infrastructural support, is crucial. The critical care community must continue to engage the necessary authorities, so as to unleash its own immense potential, and to improve long-term survival and quality of life for the critically ill.

## Conclusions

Critical care has grown at different rates across Asia’s many countries since its birth in the 1960s, and multiple challenges remain. These challenges should be addressed through a collaborative approach across disciplines, ICUs, hospitals, societies, governments, and countries.

## Supplementary Information

**Additional file 1.** Asian countries within the United Nations Asia-Pacific Regional Group.

**Additional file 2.** Questionnaire.

**Additional file 3.** Writing process for the manuscript.

**Additional file 4.** Staffing in ICUs.

**Additional file 5.** Asian member societies of the World Federation of Intensive and Critical Care.

**Additional file 6.** Critical care beds per 100,000 population.

## Data Availability

Not applicable.

## Abbreviations

ACCCT: Asian Critical Care Clinical Trials
ACME: Asian Collaboration for Medical Ethics study
APACCM: Asia Pacific Association of Critical Care Medicine
APACHE: Acute Physiology and Chronic Health Evaluation
AVF: Asia Ventilation Forum
BASIC: Basic Assessment and Support in Intensive Care Collaboration
COVID-19: Coronavirus disease 2019
EPIC III: Extended Study on Prevalence of Infection in Intensive Care III study
FCCS: Fundamental Critical Care Support Course
ICON: Intensive Care Over Nations audit
ICU: Intensive care unit
JAKOICS: Japan–Korea Intensive Care Study
JIPAD: Japanese Intensive care PAtient Database
MCCRC: Multi-professional Critical Care Review Course
MERS: Middle East respiratory syndrome
MOSAICS: Management Of Severe sepsis in Asia’s Intensive Care unitS study
MRIC: Malaysian Registry of Intensive Care
NICS: National Intensive Care Surveillance registry
NICUR: National Intensive Care Unit Repository
PRICE: Pakistan Registry of Intensive CarE
SARS: Severe acute respiratory syndrome
SARS-CoV-2: SARS coronavirus 2
SCCM: Society of Critical Care Medicine
PRoVENT-iMiC: PRactice of VENTilation in Middle-income Countries study
WFICC: World Federation of Intensive and Critical Care
WPACCM: Western Pacific Association of Critical Care Medicine
YLLs: Years of life lost

## References

[1] Bowring P (1987). What is “Asia”?. Far Eastern Econ Rev.

[2] United Nations. Department for General Assembly and Conference Management. 2021. https://www.un.org/dgacm/en/content/regional-groups. Accessed 8 Aug 2021.

[3] The World Bank. World Bank country and lending groups. 2021. https://datahelpdesk.worldbank.org/knowledgebase/articles/906519-world-bank-country-and-lending-groups. Accessed 8 Aug 2021.

[4] Adhikari NK, Fowler RA, Bhagwanjee S, Rubenfeld GD (2010). Critical care and the global burden of critical illness in adults. Lancet.

[5] Lumb PD, Adler DC, Al Rahma H, Amin P, Bakker J, Bhagwanjee S (2021). International critical care-from an indulgence of the best-funded healthcare systems to a core need for the provision of equitable care. Crit Care Med.

[6] Wunsch H (2020). The outbreak that invented intensive care. Nature.

[7] Tan BH (2015). Supporting life: the journey of intensive care in Malaysia.

[8] Prayag S (2002). ICUs worldwide: critical care in India. Crit Care.

[9] Arabi YM, Phua J, Koh Y, Du B, Faruq MO, Nishimura M (2016). Structure, organization, and delivery of critical care in Asian ICUs. Crit Care Med.

[10] Alotaibi G (2015). Status of respiratory care profession in Saudi Arabia: a national survey. Ann Thorac Med.

[11] See KC, Zhao MY, Nakataki E, Chittawatanarat K, Fang WF, Faruq MO (2018). Professional burnout among physicians and nurses in Asian intensive care units: a multinational survey. Intensive Care Med.

[12] Wang J, Hu B, Peng Z, Song H, Cai S, Rao X (2021). Prevalence of burnout among intensivists in mainland China: a nationwide cross-sectional survey. Crit Care.

[13] Hong SB, Kim HJ, Huh JW, Do KH, Jang SJ, Song JS (2014). A cluster of lung injury associated with home humidifier use: clinical, radiological and pathological description of a new syndrome. Thorax.

[14] Dobb GJ (1998). Journal of critical care and shock. Crit Care Shock.

[15] Divatia JV, Jog S (2014). Intensive care research and publication in India: quo vadis?. Intensive Care Med.

[16] Hashmi M, Beane A, Taqi A, Memon MI, Athapattu P, Khan Z (2019). Pakistan Registry of Intensive CarE (PRICE): expanding a lower middle-income, clinician-designed critical care registry in South Asia. J Intensive Care Soc.

[17] Ma P, Du B (2013). Critical care research in mainland China: more needed on the international stage. Intensive Care Med.

[18] Lee BH, Inui D, Suh GY, Kim JY, Kwon JY, Park J (2012). Association of body temperature and antipyretic treatments with mortality of critically ill patients with and without sepsis: multi-centered prospective observational study. Crit Care.

[19] Park DW, Egi M, Nishimura M, Chang Y, Suh GY, Lim CM (2016). The association of fever with total mechanical ventilation time in critically ill patients. J Korean Med Sci.

[20] Arabi YM, Al-Hameed F, Burns KEA, Mehta S, Alsolamy SJ, Alshahrani MS (2019). Adjunctive intermittent pneumatic compression for venous thromboprophylaxis. N Engl J Med.

[21] Arabi YM, Mandourah Y, Al-Hameed F, Sindi AA, Almekhlafi GA, Hussein MA (2018). Corticosteroid therapy for critically ill patients with Middle East respiratory syndrome. Am J Respir Crit Care Med.

[22] Arabi YM, Al-Omari A, Mandourah Y, Al-Hameed F, Sindi AA, Alraddadi B (2017). Critically ill patients with the Middle East respiratory syndrome: a multicenter retrospective cohort study. Crit Care Med.

[23] Bellani G, Laffey JG, Pham T, Fan E, Brochard L, Esteban A (2016). Epidemiology, patterns of care, and mortality for patients with acute respiratory distress syndrome in intensive care units in 50 countries. JAMA.

[24] Cecconi M, Hofer C, Teboul JL, Pettila V, Wilkman E, Molnar Z (2015). Fluid challenges in intensive care: the FENICE study: a global inception cohort study. Intensive Care Med.

[25] Citerio G, Prisco L, Oddo M, Meyfroidt G, Helbok R, Stocchetti N (2019). International prospective observational study on intracranial pressure in intensive care (ICU): the SYNAPSE-ICU study protocol. BMJ Open.

[26] Venkatesh B, Finfer S, Cohen J, Rajbhandari D, Arabi Y, Bellomo R (2018). Adjunctive glucocorticoid therapy in patients with septic shock. New Engl J Med.

[27] Hodgson CL, Cooper DJ, Arabi Y, King V, Bersten A, Bihari S (2019). Maximal recruitment open lung ventilation in acute respiratory distress syndrome (PHARLAP). A phase II, multicenter randomized controlled clinical trial. Am J Respir Crit Care Med.

[28] Shehabi Y, Howe BD, Bellomo R, Arabi YM, Bailey M, Bass FE (2019). Early sedation with dexmedetomidine in critically ill patients. New Engl J Med.

[29] Ferguson ND, Cook DJ, Guyatt GH, Mehta S, Hand L, Austin P (2013). High-frequency oscillation in early acute respiratory distress syndrome. N Engl J Med.

[30] Vincent JL, Marshall JC, Ñamendys-Silva SA, François B, Martin-Loeches I, Lipman J (2014). Assessment of the worldwide burden of critical illness: the Intensive Care Over Nations (ICON) audit. Lancet Respir Med.

[31] Phua J, Koh Y, Du B, Tang YQ, Divatia JV, Tan CC (2011). Management of severe sepsis in patients admitted to Asian intensive care units: prospective cohort study. BMJ.

[32] Phua J, Joynt GM, Nishimura M, Deng Y, Myatra SN, Chan YH (2015). Withholding and withdrawal of life-sustaining treatments in intensive care units in Asia. JAMA Intern Med.

[33] Phua J, Joynt GM, Nishimura M, Deng Y, Myatra SN, Chan YH (2016). Withholding and withdrawal of life-sustaining treatments in low-middle-income versus high-income Asian countries and regions. Intensive Care Med.

[34] Phua J, Faruq MO, Kulkarni AP, Redjeki IS, Detleuxay K, Mendsaikhan N (2020). Critical care bed capacity in Asian countries and regions. Crit Care Med.

[35] Phua J, Weng L, Ling L, Egi M, Lim CM, Divatia JV (2020). Intensive care management of coronavirus disease 2019 (COVID-19): challenges and recommendations. Lancet Respir Med.

[36] Leung CHC, Lee A, Arabi YM, Phua J, Divatia JV, Koh Y (2021). Mechanical ventilation discontinuation practices in Asia: a multinational survey. Ann Am Thorac Soc.

[37] Park SY, Phua J, Nishimura M, Deng Y, Kang Y, Tada K (2018). End-of-life care in ICUs in East Asia: a comparison among China, Korea, and Japan. Crit Care Med.

[38] Faruq MO, Ahsan A, Uddin M, Khatun U, Mannan MA, Tamanna R (2013). Implementation of sepsis bundles in intensive care units of Bangladesh: a prospective observational study. Bangladesh Crit Care J.

[39] Faruq MO, Nooruzzaman A, Tamanna R, Sultana A, Mallick U, Asaduzzaman M (2019). Withholding and withdrawal of life-sustaining treatments in critically ill ICU patients: a study on attitude of physicians of Bangladesh. Bangladesh Crit Care J.

[40] Faruq MO, Nooruzzaman ARM, Tamanna R, Huda AK, Sultana A, Mallick U (2019). An analysis of structure, organization and delivery of ICU care in Bangladesh. Bangladesh Crit Care J.

[41] Phua J, Ho BC, Tee A, Chan KP, Johan A, Loo S (2012). The impact of clinical protocols in the management of severe sepsis: a prospective cohort study. Anaesth Intensive Care.

[42] Wang H, Naghavi M, Allen C, Barber RM, Bhutta ZA, Carter A (2016). Global, regional, and national life expectancy, all-cause mortality, and cause-specific mortality for 249 causes of death, 1980–2015: a systematic analysis for the Global Burden of Disease Study 2015. Lancet.

[43] Rudd KE, Johnson SC, Agesa KM, Shackelford KA, Tsoi D, Kievlan DR (2020). Global, regional, and national sepsis incidence and mortality, 1990–2017: analysis for the Global Burden of Disease Study. Lancet.

[44] Vincent JL, Sakr Y, Singer M, Martin-Loeches I, Machado FR, Marshall JC (2020). Prevalence and outcomes of infection among patients in intensive care units in 2017. JAMA.

[45] Fatema K, Ahsan ASM, Barai L, Ahmed F, Haq J, Faruq MO (2016). Bacterial profile and their antibiotic resistance in an ICU of Bangladesh: comparison of four studies from 2004 to 2011. Bangladesh Crit Care J.

[46] Venkataraman R, Divatia JV, Ramakrishnan N, Chawla R, Amin P, Gopal P (2018). Multicenter observational study to evaluate epidemiology and resistance patterns of common intensive care unit-infections. Indian J Crit Care Med.

[47] Parajuli NP, Acharya SP, Mishra SK, Parajuli K, Rijal BP, Pokhrel BM (2017). High burden of antimicrobial resistance among gram negative bacteria causing healthcare associated infections in a critical care unit of Nepal. Antimicrob Resist Infect Control.

[48] Tran GM, Ho-Le TP, Ha DT, Tran-Nguyen CH, Nguyen TSM, Pham TTN (2017). Patterns of antimicrobial resistance in intensive care unit patients: a study in Vietnam. BMC Infect Dis.

[49] Southeast Asia Infectious Disease Clinical Research Network (2017). Causes and outcomes of sepsis in southeast Asia: a multinational multicentre cross-sectional study. Lancet Glob Health.

[50] Crit Care Asia (2020). Establishing a critical care network in Asia to improve care for critically ill patients in low- and middle-income countries. Crit Care.

[51] Irie H, Okamoto H, Uchino S, Endo H, Uchida M, Kawasaki T (2020). The Japanese Intensive care PAtient Database (JIPAD): a national intensive care unit registry in Japan. J Crit Care.

[52] Malaysian Registry of Intensive Care. Annual report. 2017. http://www.crc.gov.my/wp-content/uploads/documents/report/mric_report_2017.pdf. Accessed 31 May 2021.

[53] National Intensive Care Surveillance. A critical care clinical registry and bed availability system for Sri Lanka. https://nicst.com/iframe-icu-phpgrid-dev/. Accessed 31 May 2021.

[54] Park J, Jeon K, Chung CR, Yang JH, Cho YH, Cho J (2018). A nationwide analysis of intensive care unit admissions, 2009–2014 - The Korean ICU National Data (KIND) study. J Crit Care.

[55] Divatia JV, Amin PR, Ramakrishnan N, Kapadia FN, Todi S, Sahu S (2016). Intensive care in India: the Indian Intensive Care Case Mix and Practice Patterns Study. Indian J Crit Care Med.

[56] Weng L, Zeng XY, Yin P, Wang LJ, Wang CY, Jiang W (2018). Sepsis-related mortality in China: a descriptive analysis. Intensive Care Med.

[57] Srivastava A, Peshin SS, Kaleekal T, Gupta SK (2005). An epidemiological study of poisoning cases reported to the National Poisons Information Centre, All India Institute of Medical Sciences. New Delhi Hum Exp Toxicol.

[58] Rizwan M, Hashmi M, Zafar H (2018). A six-month retrospective study of resources burden by trauma victims in the surgical intensive care unit of a university hospital in Pakistan. Cureus..

[59] Nickol ME, Kindrachuk J (2019). A year of terror and a century of reflection: perspectives on the great influenza pandemic of 1918–1919. BMC Infect Dis.

[60] Potter CW (2001). A history of influenza. J Appl Microbiol.

[61] Kawana A, Naka G, Fujikura Y, Kato Y, Mizuno Y, Kondo T (2007). Spanish influenza in Japanese armed forces, 1918–1920. Emerg Infect Dis.

[62] Chandra S, Kassens-Noor E (2014). The evolution of pandemic influenza: evidence from India, 1918–19. BMC Infect Dis.

[63] World Health Organization. Summary of probable SARS cases with onset of illness from 1 November 2002 to 31 July 2003. https://www.who.int/csr/sars/country/table2004_04_21/en/. Accessed 31 May 2021.

[64] Lew TW, Kwek TK, Tai D, Earnest A, Loo S, Singh K (2003). Acute respiratory distress syndrome in critically ill patients with severe acute respiratory syndrome. JAMA.

[65] World Health Organization. MERS monthly summary, November 2019. https://www.who.int/emergencies/mers-cov/en/. Accessed 31 May 2021.

[66] Oh MD, Park WB, Park SW, Choe PG, Bang JH, Song KH (2018). Middle East respiratory syndrome: what we learned from the 2015 outbreak in the Republic of Korea. Korean J Intern Med.

[67] Liew MF, Siow WT, MacLaren G, See KC (2020). Preparing for COVID-19: early experience from an intensive care unit in Singapore. Crit Care.

[68] Wu Z, McGoogan JM (2020). Characteristics of and important lessons from the coronavirus disease 2019 (COVID-19) outbreak in China: summary of a report of 72314 cases from the Chinese Center for Disease Control and Prevention. JAMA.

[69] Xie J, Tong Z, Guan X, Du B, Qiu H, Slutsky AS (2020). Critical care crisis and some recommendations during the COVID-19 epidemic in China. Intensive Care Med.

[70] Hua J, Qian C, Luo Z, Li Q, Wang F (2020). Invasive mechanical ventilation in COVID-19 patient management: the experience with 469 patients in Wuhan. Crit Care.

[71] Lancet T (2021). India’s COVID-19 emergency. Lancet.

[72] Anand A, Sandefur J, Subramanian A. Three new estimates of India’s all cause excess mortality during the COVID-19 pandemic. Center for Global Development. 2021. https://www.cgdev.org/sites/default/files/three-new-estimates-indias-all-cause-excess-mortality-during-covid-19-pandemic.pdf. Accessed 11 August 2021.

[73] Mallapaty S (2021). Delta threatens rural regions that dodged earlier COVID waves. Nature.

[74] World Health Organization. WHO coronavirus (COVID-19) dashboard. 2021. https://covid19.who.int/table. Accessed 11 August 2021.

[75] Kwon S (2011). Health care financing in Asia: key issues and challenges. Asia Pac J Public Health.

[76] Rahman MM, Khanam R, Rahman M (2018). Health care expenditure and health outcome nexus: new evidence from the SAARC-ASEAN region. Global Health.

[77] Pisani L, Algera AG, Serpa Neto A, Ahsan A, Beane A, Chittawatanarat K (2021). Epidemiological characteristics, ventilator management, and clinical outcome in patients receiving invasive ventilation in intensive care units from 10 Asian middle-income countries (PRoVENT-iMiC): an international, multicenter, prospective study. Am J Trop Med Hyg.

[78] Abe T, Kushimoto S, Tokuda Y, Phillips GS, Rhodes A, Sugiyama T (2019). Implementation of earlier antibiotic administration in patients with severe sepsis and septic shock in Japan: a descriptive analysis of a prospective observational study. Crit Care.

[79] He H, Ma X, Su L, Wang L, Guo Y, Shan G (2020). Effects of a national quality improvement program on ICUs in China: a controlled pre-post cohort study in 586 hospitals. Crit Care.

[80] Good care of the dying patient (1996). Council on Scientific Affairs, American Medical Association. JAMA.

[81] Chan HM (2004). Sharing death and dying: advance directives, autonomy and the family. Bioethics.

[82] Kwak J, Haley WE (2005). Current research findings on end-of-life decision making among racially or ethnically diverse groups. Gerontologist.

[83] Vollset SE, Goren E, Yuan CW, Cao J, Smith AE, Hsiao T (2020). Fertility, mortality, migration, and population scenarios for 195 countries and territories from 2017 to 2100: a forecasting analysis for the Global Burden of Disease Study. Lancet.

[84] Phua J, Hashmi M, Haniffa R (2020). ICU beds: less is more? Not sure. Intensive Care Med.

[85] Arabi YM, Azoulay E, Al-Dorzi HM, Phua J, Salluh J, Binnie A (2021). How the COVID-19 pandemic will change the future of critical care. Intensive Care Med.

[86] Vincent JL, Wendon J, Martin GS, Juffermans NP, Creteur J, Cecconi M (2021). COVID-19: what we’ve done well and what we could or should have done better-the 4 Ps. Crit Care.

[87] Dondorp AM, Iyer SS, Schultz MJ (2016). Critical care in resource-restricted settings. JAMA.

