# Supplementary material for: The story of critical care in Asia: a narrative review
Source: J Intensive Care. 2021 Oct 7;9:60. doi: 10.1186/s40560-021-00574-4 (PMC8496144; doi:10.1186/s40560-021-00574-4)
Supplement: Supplementary file 1 — Additional file 1. Asian countries within the United Nations Asia-Pacific Regional Group. [file 40560_2021_574_MOESM1_ESM.docx]

**Additional File 1** Asian countries within the United Nations Asia-Pacific Regional Group^a^

| **Low-income^b^** | **Middle-income^b^** | | **High-income^b^** |
| --- | --- | --- | --- |
|  | **Lower-middle** | **Upper-middle** |  |
| Afghanistan | Bangladesh | China | Bahrain |
| North Korea | Bhutan | Iraq | Brunei |
| Yemen | Cambodia | Jordan | Cyprus |
| Syria | India | Kazakhstan | Hong Kong^c^ |
|  | Indonesia | Lebanon | Japan |
|  | Iran | Malaysia | Kuwait |
|  | Kyrgyzstan | Maldives | Oman |
|  | Laos | Thailand | Qatar |
|  | Mongolia | Turkmenistan | Saudi Arabia |
|  | Myanmar |  | Singapore |
|  | Nepal |  | South Korea |
|  | Pakistan |  | Taiwan^c^ |
|  | Philippines |  | United Arab Emirates |
|  | Sri Lanka |  |  |
|  | Tajikistan |  |  |
|  | Timor-Leste |  |  |
|  | Uzbekistan |  |  |
|  | Vietnam |  |  |

^a^ Use of the list from the United Nations Asia-Pacific Regional Group excludes countries which while sited at least in part in Asia are sometimes considered to be part of Europe or Africa, and the Middle East, such as Armenia, Azerbaijan, Egypt, Israel, Palestine, Russia, and Turkey [2]

^b^ According to the 2021-2022 World Bank income classification, which uses gross national income per capita in 2020 [3]. Note that changes in the countries’ classification across the years are not unusual

^c^ Hong Kong and Taiwan are classified separately due to their different economic and healthcare systems
