# Supplementary material for: The story of critical care in Asia: a narrative review
Source: J Intensive Care. 2021 Oct 7;9:60. doi: 10.1186/s40560-021-00574-4 (PMC8496144; doi:10.1186/s40560-021-00574-4)
Supplement: Supplementary file 2 — Additional file 2. Questionnaire. [file 40560_2021_574_MOESM2_ESM.docx]

**Additional File 2** Questionnaire

**COUNTRY AND NATIONAL COORDINATOR**

**Question 1**

Name of country

|  |
| --- |

**Question 2**

Name of national coordinator and academic qualifications of national coordinator (e.g. MD, PhD, MBBS, MRCP, FCCP, etc)

|  |
| --- |

**Question 3**

Hospital affiliation of national coordinator

|  |
| --- |

**Question 4**

Relationship of your intensive/critical care society/societies with national coordinator (e.g. current or past president, secretary general, board member, etc)

|  |
| --- |

**Question 5**

Email address of national coordinator

|  |
| --- |

**THE EVOLUTION OF CRITICAL CARE IN YOUR COUNTRY**

**Question 6**

How did critical care start in your country? For example, when and how did the concept of a unit meant for critically ill patients come about? Only if relevant, please feel free to relate this to developments in Asia and the West, including the United States’ Society of Critical Care Medicine (SCCM) and its formation in 1970.

| (Where appropriate and available, please provide references or websites) |
| --- |

**Question 7**

Would you like to share any interesting stories or anecdotes of setbacks in the setting up or evolution of critical care in your country? These could be, for example, about heroines and heroes; failures and things that just went wrong; moves that led to blind alleys, dead ends, and U-turns.

| (Optional)  (Where appropriate and available, please provide references or websites) |
| --- |

**Question 8**

Would you like to share any interesting stories or anecdotes of successes in the setting up or evolution of critical care in your country? These could be, for example, about heroines and heroes; “Aha” moments, when enlightening realisations were made; chance encounters, where things just seemed to randomly happen by chance and yet resulted in good outcomes.

| (Optional)  (Where appropriate and available, please provide references or websites) |
| --- |

**CRITICAL CARE SOCIETIES PAST AND PRESENT IN YOUR COUNTRY**

**Question 9**

How many critical care societies are there in your country? Please name them and also state the year(s) they were formed, or let us know if there are none.

| (Where appropriate and available, please provide references or websites) |
| --- |

**Question 10**

Would you like to share any major contributions of your country’s critical care society/societies, past and present? Examples would include (but are not limited to) enhancing the quality of critical care, coping with national disasters or epidemics, helping critical care stand out as an important and independent medical specialty, contributions to public health, etc. Would you like to share any interesting stories or anecdotes about the critical care society/societies or individuals related to the society/societies?

| (Optional)  (Where appropriate and available, please provide references or websites) |
| --- |

**Question 11**

Please state if your country’s critical care society/societies are represented in international societies such as the Asia Pacific Association of Critical Care Medicine (APACCM), the World Federation of Societies of Intensive and Critical Care Medicine (WFSICCM), or any other international societies. Please provide any details which, you deem, are important.

| (Optional)  (Where appropriate and available, please provide references or websites) |
| --- |

**Question 12**

Please describe any collaborations your country’s critical care society/societies have had, are having, or will be having with international societies such as the APACCM, WFSICCM, SCCM, European Society of Intensive Care Medicine (ESICM), Australian and New Zealand Intensive Care Society (ANZICS), neighbouring societies, and any others. Please share how, if any, collaborating with other societies have had a synergistic effect on system improvement, clinical performance, education, or research.

| (Optional)  (Where appropriate and available, please provide references or websites) |
| --- |

**Question 13**

**Please answer this question only if your country does not have** a critical care society: how does your country champion critical care and collaborate without a critical care society?

| (Optional)  (Where appropriate and available, please provide references or websites) |
| --- |

**ROLE OF GOVERNMENT IN CRITICAL CARE IN YOUR COUNTRY**

**Question 14**

How supportive is your government for the development of critical care in your country/region? Please tick one.

Very supportive

Supportive

Neither supportive nor unsupportive

Unsupportive

Very unsupportive

**CRITICAL CARE EDUCATION IN YOUR COUNTRY**

**Question 15**

In what year did critical care become a recognised specialty in your country? Please state if this is recognised by your government or just by an academic society or both. Please state if critical care is not a recognised specialty.

| (Where appropriate and available, please provide references or websites) |
| --- |

**Question 16**

In what year was an accredited training programme for critical care created for doctors in your country? Please state if there is no accredited training programme.

| (Where appropriate and available, please provide references or websites) |
| --- |

**Question 17**

In what year was an accredited training programme for critical care created for nurses in your country? Please state also if there is no accredited training programme.

| (Where appropriate and available, please provide references or websites) |
| --- |

**Question 18**

In what year was an accredited training programme created for respiratory therapists in your country? Please state if there are respiratory therapists but no accredited training programme, or if there no respiratory therapists.

| (Where appropriate and available, please provide references or websites) |
| --- |

**CRITICAL CARE RESEARCH IN YOUR COUNTRY**

**Question 19**

How strong is the support, infrastructure, and funding for critical care research in your country? Please tick one.

Very strong

Strong

Neither strong nor weak

Weak

Very weak

**Question 20**

How many multicentre critical care research groups are there in your country? This refers only to groups that continue to collaborate beyond one project or programme (an example is the ANZICS Clinical Trials Group). Please name them and also state the year(s) they were formed, or let us know if there are none.

| (Where appropriate and available, please provide references or websites) |
| --- |

**Question 21**

Please describe any collaborations your country has had, is having, or will be having with international critical care research societies such as the Asian Critical Care Clinical Trials (ACCCT) Group, ESICM, ANZICS Clinical Trials Group, and any others.

| (Optional)  (Where appropriate and available, please provide references or websites) |
| --- |

**Question 22**

Would you like to share any success stories or difficult challenges your country has or had for critical care research? Please feel free to name some heroes or heroines if appropriate.

| (Optional)  (Where appropriate and available, please provide references or websites) |
| --- |

**EPIDEMIOLOGY OF CRITICAL ILLNESS IN YOUR COUNTRY**

**Question 23**

Would you like to cite some publications (international or domestic journals) on the epidemiology of critical illness in your country?

| (Optional)  (Where appropriate and available, please provide references or websites) |
| --- |

**Question 24**

Would you like to share any success stories or difficult challenges your country has or had in understanding the epidemiology of critical illness in your country? Please feel free to name some heroes or heroines if appropriate.

| (Optional)  (Where appropriate and available, please provide references or websites) |
| --- |

**ACCESSIBILITY AND COSTS OF CRITICAL CARE IN YOUR COUNTRY**

**Question 25**

Would you like to cite some publications (international or domestic journals) on how accessible and/or costly critical care is to the people in your country? Accessibility refers to how easy or difficult it is for patients to receive critical care. Costs can be seen from the patients’ and/or the providers’ perspectives.

| (Optional)  (Where appropriate and available, please provide references or websites) |
| --- |

**Question 26**

Would you like to share any success stories or difficult challenges your country has or had on accessibility and costs of critical care for the people in your country? Accessibility refers to how easy or difficult it is for patients to receive critical care. Costs can be seen from the patients’ or the providers’ perspectives. Please feel free to name some heroes or heroines if appropriate.

| (Optional)  (Where appropriate and available, please provide references or websites) |
| --- |

**QUALITY OF CRITICAL CARE IN YOUR COUNTRY**

**Question 27**

Would you like to cite some publications (international or domestic journals) on the quality of critical care in your country?

| (Optional)  (Where appropriate and available, please provide references or websites) |
| --- |

**Question 28**

Would you like to share any success stories or difficult challenges your country has or had on the quality of critical care in your country? Please feel free to name some heroes or heroines if appropriate.

| (Optional)  (Where appropriate and available, please provide references or websites) |
| --- |

**THE FUTURE OF CRITICAL CARE IN YOUR COUNTRY**

**Question 29**

Would you like to predict how critical care in your country will look like in 2050? Would you like to share what your country is doing to get there, including the critical care community, societies, research groups, and government, etc?

| (Optional)  (Where appropriate and available, please provide references or websites) |
| --- |

**CRITICAL CARE BED CAPACITY IN YOUR COUNTRY**

**Questions 30 to 34 are only for countries that did not take part in the Asian Assessment of Bed Capacity (ABC) Study.**

**Question 30**

Please let us know how many intensive care units (ICUs) there are in your country, and please state the year that this information applies to. Please also state if data are not available.

| (Where appropriate and available, please provide references or websites) |
| --- |

**Question 31**

Please let us know how many ICU beds there are in your country, and please state the year that this information applies to. Please also state if data are not available.

| (Where appropriate and available, please provide references or websites) |
| --- |

**Question 32**

Please let us know how many intermediate care units (IMCUs, variously known as intermediate care areas, high-dependency units, step-up units, and step-down units) there are in your country, and please state the year that this information applies to. Please also state if data are not available.

| (Where appropriate and available, please provide references or websites) |
| --- |

**Question 33**

Please let us know how many IMCU beds there are in your country, and please state the year that this information applies to, please also state if data are not available.

| (Where appropriate and available, please provide references or websites) |
| --- |

**ETHICAL ISSUES IN CRITICAL CARE IN YOUR COUNTRY**

**Question 34**

Please let us know what you consider common ethical issues of critical care practices in your country.

Please tick three common issues.

End-of-life care related issues

Patient autonomy and respect issues (such as dominating family overriding patient’s own decisions)

Informed consent related issues

Inappropriate behaviour of healthcare professional (such as not responding to a nurse call)

Resource allocation related issues (including triage issues)

Organ transplantation related issues

Discharge against medical advice (discontinuation of treatment because of medical bill burden in critically ills, who seems to have high chance of recovery)

Patient transportation related issues (such as uninformed transfer from/to other hospital)

Communication related issues between caregivers

Communication related issues between caregivers and patient/patient’s family

Gender discrimination issues

Racial discrimination issues

Futility issues between caregivers and patient/patient’s family

Conflict of interest related issues (such as between caregivers and pharmaceutical company or medical device company)

Clinical research related issues

Others – please describe in box below:

| (Optional)  (Where appropriate and available, please provide references or websites) |
| --- |

Thanks for your effort in replying to these long and difficult questions.
