# Supplementary material for: The story of critical care in Asia: a narrative review
Source: J Intensive Care. 2021 Oct 7;9:60. doi: 10.1186/s40560-021-00574-4 (PMC8496144; doi:10.1186/s40560-021-00574-4)
Supplement: Supplementary file 3 — Additional file 3. Writing process for the manuscript. [file 40560_2021_574_MOESM3_ESM.docx]

**Additional File 3** Writing process for the manuscript

To write this review the co-authors, all prominent intensivists from the Asian Critical Care Clinical Trials (ACCCT) Group who represented 19 countries and regions, some of whom are current or past Presidents of their national critical care societies, first provided qualitative input through a questionnaire (Additional File 2). Selected sections of the original text were featured in a recently published article commemorating the Society of Critical Care Medicine’s 50th anniversary “International critical care – from an indulgence of the best funded health care systems to a core need for the provision of equitable care” [5]. The manuscript has since been substantially expanded and updated for both breadth and depth.

Additional information on Asian critical care and beyond that featured in the Society of Critical Care Medicine manuscript include:

- Timelines for the development of intensive care units (ICUs) and critical care as a specialty
- Timelines for the development of accredited training programmes for intensivists, critical care nurses, and respiratory therapists
- Number and shortage of critical care healthcare workers beyond doctors and nurses
- Lists of critical care societies and scientific congresses
- Selected national and international multicentre critical care research groups
- Epidemiology of critical illness
- Details of past epidemics and pandemics
- Details of the current coronavirus disease 2019 (COVID-19)
- Number of critical care beds per 100,000 population per country
- How medical bill size and costs affect goals of care
- Findings from several international studies on the quality of critical care
- Patient autonomy versus the opinions of the family in end-of-life care
- Factors driving demand and ensuring supply for critical care
- Enablers of good outcomes
