# Supplementary material for: The story of critical care in Asia: a narrative review
Source: J Intensive Care. 2021 Oct 7;9:60. doi: 10.1186/s40560-021-00574-4 (PMC8496144; doi:10.1186/s40560-021-00574-4)
Supplement: Supplementary file 4 — Additional file 4. Staffing in ICUs. [file 40560_2021_574_MOESM4_ESM.docx]

**Additional File 4** Staffing in ICUs


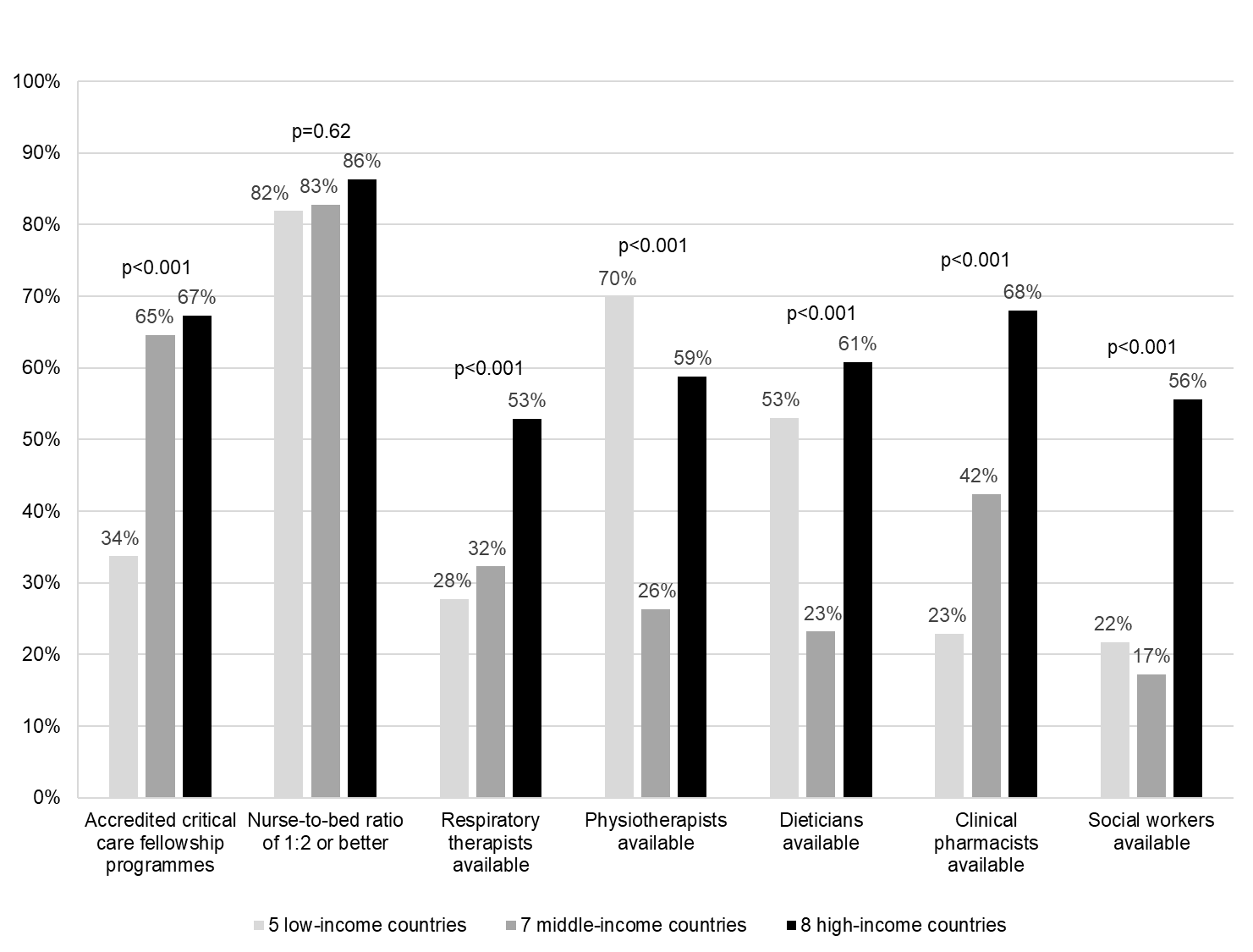


*ICU*, intensive care unit

Data from the Asian ICUs Structure and Process (AISP) study of 335 ICUs in 20 Asian countries, conducted between 2013 and 2014 [9]. Countries are categorised according to the World Bank income classification. p-values refer to unadjusted statistical comparisons using the chi-square test. This survey involved many large government referral hospitals, and it is likely that smaller ICUs scattered across low-incomes countries are even more resource-limited
