# Supplementary material for: The story of critical care in Asia: a narrative review
Source: J Intensive Care. 2021 Oct 7;9:60. doi: 10.1186/s40560-021-00574-4 (PMC8496144; doi:10.1186/s40560-021-00574-4)
Supplement: Supplementary file 5 — Additional file 5. Asian member societies of the World Federation of Intensive and Critical Care. [file 40560_2021_574_MOESM5_ESM.docx]

**Additional File 5** Asian member societies of the World Federation of Intensive and Critical Care

| **Societies** |
| --- |
| Bangladesh Society of Critical Care Medicine |
| Chinese Society of Critical Care Medicine |
| College of Anaesthesiologists and Intensivists of Sri Lanka |
| Hong Kong Society of Critical Care Medicine |
| Indian Society of Critical Care Medicine |
| Indonesian Society of Intensive Care Medicine |
| Japanese Society of Intensive Care Medicine |
| Korean Society of Critical Care Medicine |
| Kuwait Society of Critical Care |
| Lebanese Society of Critical Care Medicine |
| Malaysian Society of Intensive Care |
| Mongolian Society of Anaesthesiology and Intensive Care Physicians |
| Mongolian Society of Intensive Care Medicine |
| Nepalese Society of Critical Care Medicine |
| Oman Society of Anaesthesia and Critical Care |
| Pakistan Society of Intensive Care |
| Pakistan Society of Critical Care Medicine |
| Philippine Society of Anesthesiologists |
| Philippine Society of Critical Care Medicine |
| Saudi Critical Care Society |
| Society of Critical Care Medicine of the Chinese Association of Integration of Traditional Medicine & Modern Medicine |
| Society of Intensive Care Medicine (Singapore) |
| Sri Lankan Society of Critical Care and Emergency Medicine |
| Taiwan Society of Critical Care Medicine |
| Taiwan Society of Emergency and Critical Care Medicine |
