# Supplementary material for: The story of critical care in Asia: a narrative review
Source: J Intensive Care. 2021 Oct 7;9:60. doi: 10.1186/s40560-021-00574-4 (PMC8496144; doi:10.1186/s40560-021-00574-4)
Supplement: Supplementary file 6 — Additional file 6. Critical care beds per 100,000 population. [file 40560_2021_574_MOESM6_ESM.docx]

**Additional File 6** Critical care beds per 100,000 population


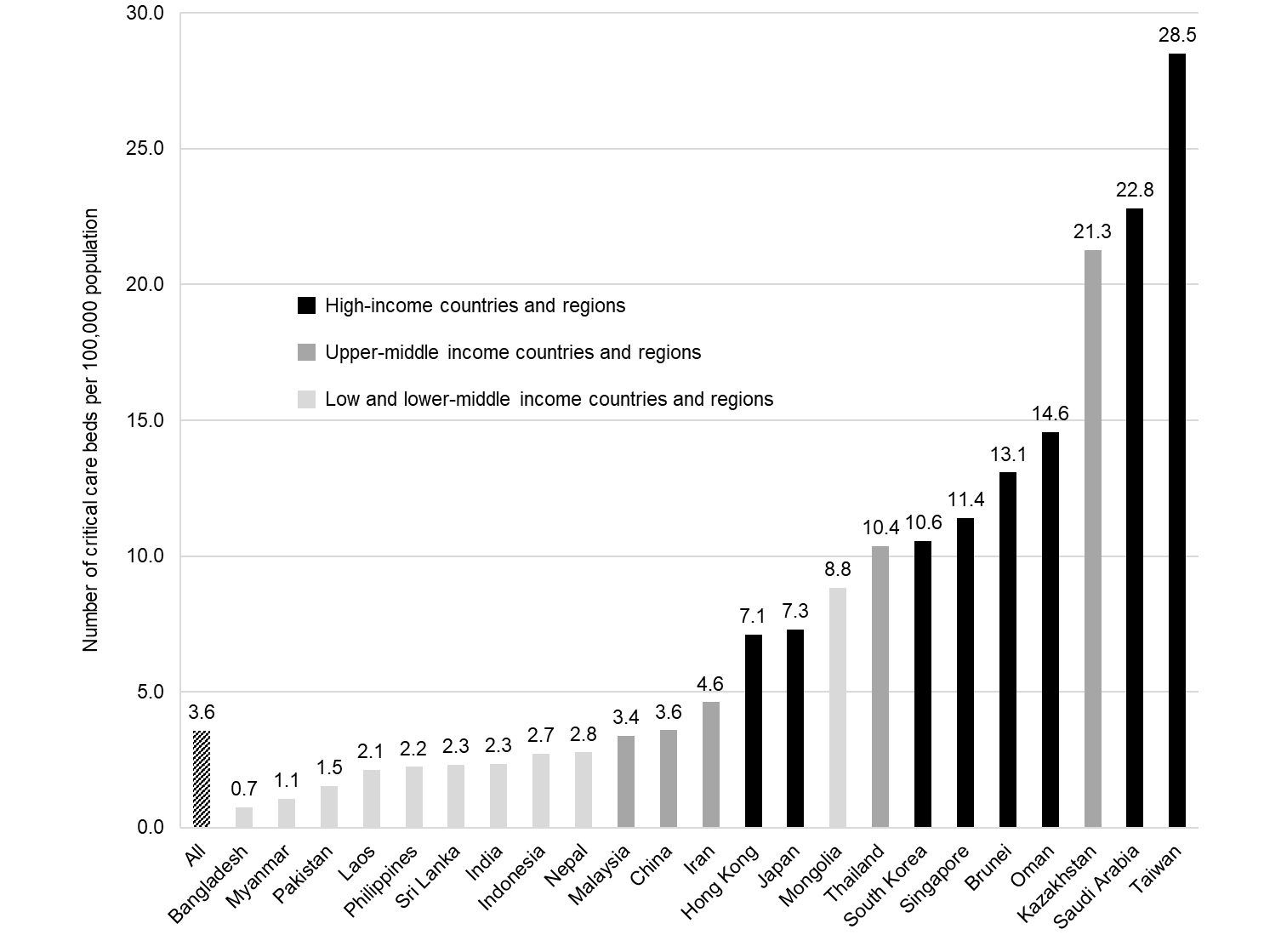


*ICU*, intensive care unit

Data from the Asian Analysis of Bed Capacity in Critical Care (ABC) Study of 23 Asian countries and regions, conducted between 2017 and 2019 [34]. Countries are categorised according to the World Bank income classification. Critical care beds refer to both intensive care units (ICUs) and intermediate care units (IMCUs) (high dependency units). Data on the number of IMCU beds in Indonesia, India, Laos, Malaysia, Pakistan, Saudi Arabia, and Thailand are not available.
